# The Role of Academia in Data Science Education

**DOI:** 10.1162/99608f92.dd363929

**Published:** 2020-01-31

**Authors:** Rafael A. Irizarry

**Affiliations:** 1 Department of Data Sciences, Dana-Farber Cancer Institute, Harvard Medical School, Harvard University, Boston, Massachusetts, United States of America; 2 Department of Biostatistics, T.H. Chan School of Public Health, Harvard University, Cambridge, Massachusetts, United States of America

**Keywords:** applied statistics, data science, data science curriculum, data wrangling, machine learning, software engineering

## Abstract

As the demand for data scientists continues to grow, universities are trying to figure out how to best contribute to the training of a workforce. However, there does not appear to be a consensus on the fundamental principles, expertise, skills, or knowledge-base needed to define an academic discipline. We argue that data science is not a discipline but rather an umbrella term used to describe a complex process involving not one data scientist possessing all the necessary expertise, but a team of data scientists with nonoverlapping complementary skills. We provide some recommendations for how to take this into account when designing data science academic programs.

## 1. What Is Data Science and Why Do We Need a New Term?

To discuss data science education, we first need to understand what it is. In the first issue of the *Harvard Data Science Review* (*HDSR*), Jeannette Wing defines data science as “the study of extracting value from data” (Wing, 2019). This is similar to the American Statistical Association’s definition of Statistics: “the science of learning from data and of measuring, controlling, and communicating uncertainty,”^1^ which raises the question, *do we need a new term?* And if so, *what is the definition?* Looking through the pages of academic Data Science Initiatives,^2^ reveals a lack of consensus. Most definitions are incongruent and fail to provide a consensus on describing the fundamental principles, expertise, skills, or knowledge-base shared by data scientists. If universities are going to offer data science degrees, they will have to define a core curriculum. Providing a detailed definition, that justifies the need for a new term, is an important first step. Here we offer an attempt.

**Figure 1.**
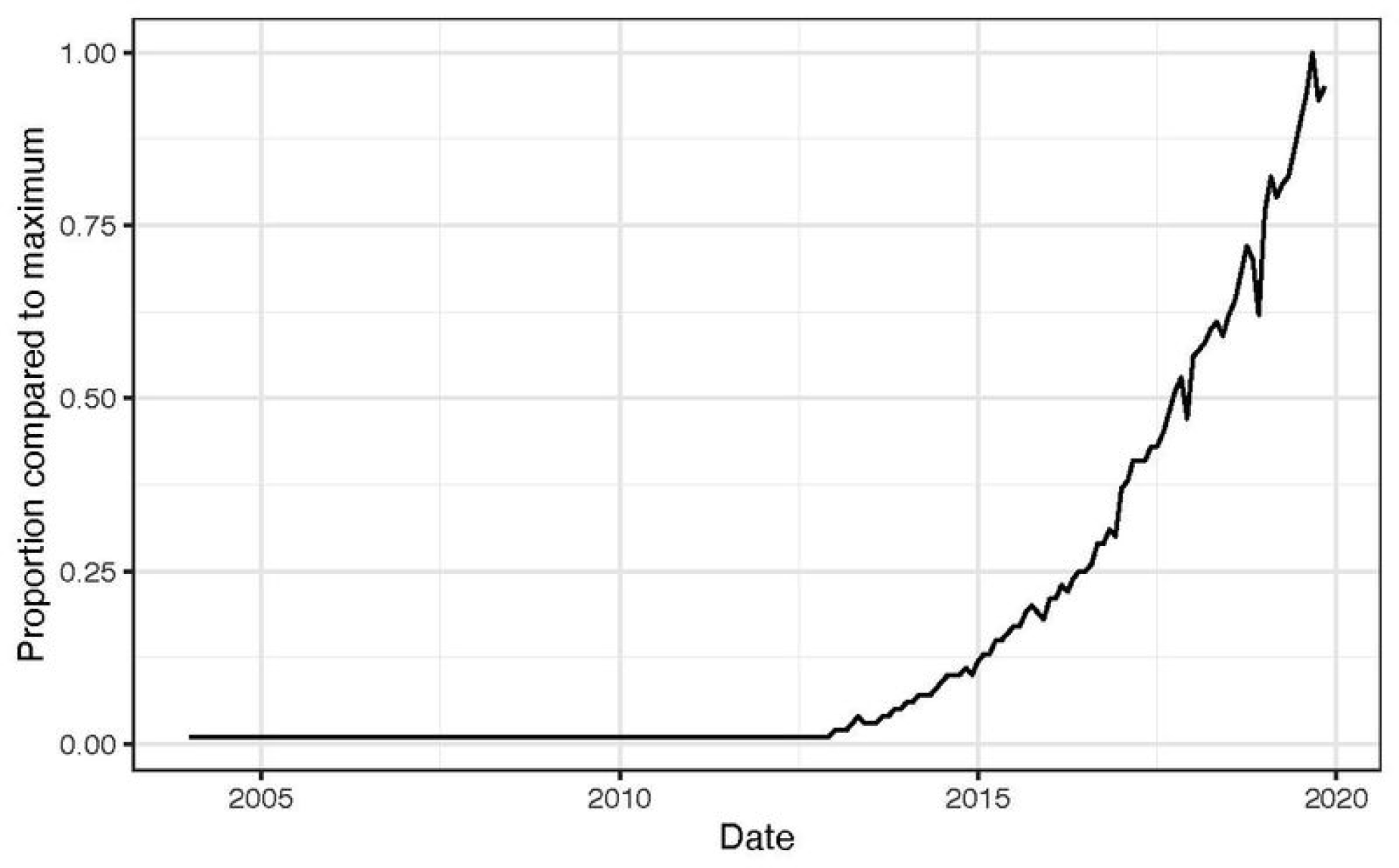
Google Trends monthly data for the term “Data Science” as of November 30, 2019. The y-axis is the proportions of searches compared to the maximum, which was on September 2019.

Although the term *data science* was originally coined in academia, the proliferation of its use has been mostly driven by the tech industry. Academics, motivated by the need to formally include data analysis and computing as academic endeavors, have used the term since at least the 1960s (Naur, 1966). In 1997, Jeff Wu, the chair of a Department of Statistics, proposed changing the discipline’s name from Statistics to Data Science,^3^ and in 2001 William Cleveland published an article titled “*Data Science: An Action Plan for Expanding the Technical Areas of the Field of Statistics*” (Cleveland, 2001). However, the increase in the popularity of the term (Figure 1) coincides, not with these early attempts at defining a new term, but with the publication by *Harvard Business Review* of “*Data Scientist: The Sexiest Job of the 21st Century*” (Davenport & Patil, 2012). In fact, Patil, one of the authors, claims to have coined this term in 2008 while working at LinkedIn, showing a disconnect between industry and the academic literature using the term in earlier decades. As a clear distinction from the earlier proposals, in the *Harvard Business Review* publication, the authors emphasized data wrangling, the ability to “bring structure to large quantities of formless data and make analysis possible,” (p.73) rather than formal data analysis.

From this *Harvard Business Review* article, and other accounts, it is clear that during the 2000s a new type of project was becoming more and more common in the tech industry: extracting value from messy, complex, and large datasets. At first, enterprising current employees, with strong quantitative skills, trained themselves to get the job done for these companies. Unable to connect the tasks they were performing to an existing trade or academic discipline, some of these individuals commenced using the term *data scientists.* We note that, at the time, similar data-driven initiatives were commonplace in academia as well, for example, in astrophysics (Zhang & Zhao, 2015), particle physics (Antcheva et al., 2009), and genomics (ENCODE Profject Consortium, 2004; Weinstein et al., 2013). However, the term *data scientist* was not used by these academic groups, perhaps because data wrangling and analysis were viewed as part of what astrophysics, particle physics, and computational biologist teams do. The term thus remained very much associated with the tech industry.

As the demand in employees capable of completing data-driven projects increased, the term *data scientist* quickly became particularly prominent because it helped recruiters specify the type of employee they wanted. Postgraduate degrees in the two disciplines most associated with data analysis and management, Statistics and Computer Science, did not guarantee the expertise needed to successfully complete these projects.

Programming skills and experience organizing and analyzing messy, complex, and large datasets were fundamental. But because you can write a Statistics Ph.D. thesis focusing, for example, on mathematically proving that estimators are asymptotically normal without ever looking at a real dataset, *statistician* was not a specific enough job title. And because you can write a Computer Science Ph.D. thesis focusing on mathematically proving problems are NP-complete without writing code or accessing databases, *computer scientist* was not specific enough either. Statisticians and computer scientists could definitely be good hires, but not always. As a result, the credentials provided by universities did not provide a very useful signal to these employers. The term *data scientist* therefore became useful for making the distinction between, for example, those with experience organizing and analyzing data, in all its messy glory, by writing fast, efficient, interoperable, and reliable code, from those with more mathematical or theoretical expertise.

However, today there is much more diversity in the challenges posed by data-driven enterprises than there was 15 years ago. The need for data scientists has expanded well beyond the tech industry to other sectors, including government (Potok, 2019). With the type of problems varying greatly across different organizations, and even within organizations, the term *data science* remains quite vague. In fact, in the first editorial summary of the *Harvard Data Science Review*, Meng (2019a) discussed why data science “is not even a single discipline by itself” and reported that the increasing consensus that the term data scientist is only useful as an umbrella term. With this in mind and taking into account that the majority of industry and government recruiters searching for data scientists are not interested in ‘the study,’ but in the extraction of value itself, we adapt Wing’s definition and propose that ‘data science is an umbrella term to describe the entire complex and multistep processes used to extract value from data.’ As described in the following, under this definition, data science includes several areas of expertise and we should not expect one individual to encompass all of these.

## 2. The Data Science Areas of Expertise

To describe what falls under the data science umbrella, we first make one big distinction between backend and frontend data science.^4^ We define the backend as the part that deals with hardware, efficient computing, and data storage infrastructure, or what is often referred to as data engineering.^5^ We define the frontend as the part geared more toward data analysis and can be further divided into tasks performed by data analysts and machine learning engineers. The data analysts wrangle, explore, quality assess, fit models to data, perform statistical inference, and develop protypes. The machine learning engineers build and assess prediction algorithms and make the solution scalable and robust for many users. Domain knowledge is of course important for both these tasks (Berthold, 2019). Often, to finish the project the backend engineers integrate a final solution into a robust automatized pipeline.

Another area of expertise for data science is defined by what we call the data science software developers. These experts are not necessarily involved directly in producing data science pipelines but instead develop the software tools that facilitate data science. They tend to have experience in frontend data science and backend engineering skills and use this experience and their skills to develop tools that benefit many others. Examples are the developers of Hadoop, R, RStudio, IPython notebooks, TensorFlow, D3, pandas, and the tidyverse, to name a few. Because academia tends to favor methodologists^6^ over software developers, this group tends to work outside of academia, with exceptions, and often prefer being labeled software engineers or data scientists, even if they have a Ph.D. in Statistics or Computer Science. Although this is a small group in terms of numbers, their impact on the field overall is tremendous. The 2019 Committee of Presidents of Statistical Societies (COPSS) award to Hadley Wickham, Chief Scientist at RStudio, is a welcoming sign that the academia is finally recognizing such impact.

## 3. The Implication for Academic Programs

Having the goal of training an individual to be an expert that can tackle all the challenges involved in the data science process is too ambitious. However, as the term data science became more and more fashionable, demand for data science education increased accordingly. Universities rushed to figure out how to meet this demand. Developing revenue-generating master’s programs was the first priority and, as a result, today we have dozens of universities offering these degrees. But what exactly are these students being prepared to do? What do these new programs offer that existing ones did not? Given that, with some exceptions, no new faculty were hired when creating these new programs and, in many cases, few or no new classes were developed, it is not clear that a master’s degree in data science, as currently offered, provides the signal employers are looking for. In fact, it should be noted that, given the independence and project ownership required for many data science jobs, recruiters typically explicitly look for Ph.Ds.

Clearly, existing academic courses provide excellent ways of gaining some of the expertise listed above. These include courses on discrete math, probability, statistical inference and modeling, computer programming, software engineering principles, machine learning, and ethics. But this was true before data science programs emerged, or even before the term data scientist was much recognized as a job title. So, what can academia do to better prepare students for the data science workforce and to provide a better signal to industry? Here are my recommendations.

### Three different tracks.

Realize that data science is an umbrella term and be careful with the use of the term data scientist (Meng, 2019a). Instead, offer specific tracks targeted at the different aspects of data science: data engineer, data analyst, and machine learning engineer. Three tracks might still not be enough, but one certainly is not.

### Bring applications to the forefront.

Adapt statistics and machine learning courses to have applications in the forefront rather than a theoretical focus (Hicks & Irizarry, 2018). Furthermore, underscore the necessity to understand the context or subject matter of the problems. Ensure that Computer Science courses on algorithms, optimization, or data structures focus on implementations.

### Real-world experience.

Develop capstone project courses defined by open-ended questions requiring data wrangling or data collection to complete (National Academies of Sciences, Engineering, and Medicine, 2018). Capstone project courses on developing software packages will be particularly useful for those interested in becoming data science software developers. Stress the importance of reliable and reproducible code: data science teams have to produce data processing pipelines that work for many users and one needs training to learn this.

### Practical programming skills.

Courses should require students to learn and use appropriate language for the expertise they are focusing on. For example, R for data exploration and prototyping, Python, Spark, Keras, and TensorFlow for machine learning engineering, and low-level languages, such a C++, for building infrastructure. Given the ubiquity of databases, all students should be at least familiar with SQL. Programs should assure that they stay current since better tools are likely to continue to be developed.

### Focus on graduate-level programs.

Given the level of practical experience needed to become a data scientist, we recommend that data science degrees be conferred at the master’s or Ph.D. levels. An undergraduate education that includes discrete mathematics, probability, statistics, machine learning, and programming courses will prepare students for the frontend tracks. An undergraduate education that includes computer science and software engineering courses will prepare students for the backend tracks (National Academies of Sciences, Engineering, and Medicine, 2018).

Following these recommendations will require substantial investment for most universities because they do not currently employ enough faculty capable of training students in these areas. Specifically, they will have to invest faculty with real-world data science experience and invest in curriculum development (Meng, 2019b).

## Footnotes

1.
https://www.amstat.org/ASA/Newsroom.aspx

2. Some examples: https://data.berkeley.edu/academics/undergraduate-programs/data-science-major, https://datascience.harvard.edu/about, https://datascience.columbia.edu/master-of-science-in-data-science, https://mcds.cs.cmu.edu/learn-us, https://sdsi.stanford.edu/, https://midas.umich.edu/dsi/

3.
http://www2.isye.gatech.edu/~jeffwu/presentations/datascience.pdf

4.
https://www.linkedin.com/pulse/one-data-science-job-doesnt-fit-all-elena-grewal/

5.
https://hbr.org/2018/08/what-data-scientists-really-do-according-to-35-data-scientists

6. By methodologists we refer to those that develop publishable theoretical abstractions that permit data analysis ideas to be applied more widely than its original application, rather than software.
